# Supplementary material for: Research on multi-sensor fusion architecture for highway area hazard monitoring based on UAVs
Source: PLoS One. 2026 May 7;21(5):e0348241. doi: 10.1371/journal.pone.0348241 (PMC13152189; doi:10.1371/journal.pone.0348241)
Supplement: Attachment — Submitted filename: Response to Reviewers.docx [file pone.0348241.s002.docx]

**Response to Reviewers**

**Additional Editor Comments**

1．**The study focuses on five common sensors, but there are several other sensors available for UAV-based monitoring (e.g., hyperspectral, thermal infrared multispectral). A broader evaluation of sensor options would strengthen the study.**

Response: Thank you for your valuable suggestion. We highly appreciate it and have carefully studied your advice regarding expanding the scope of sensor evaluation. The decision to focus on these five types of sensors in this paper was primarily based on considerations of both research objectives and engineering feasibility, as detailed below:

This study aims to provide precise, efficient, and highly adaptable technical solutions for monitoring critical hazards such as highway slope instability and pavement collapse. The selected sensors—RGB cameras, thermal infrared imagers, multispectral sensors, LiDAR, and SAR—are currently the most widely applied, technologically mature, and practically deployable mainstream types in UAV-based highway monitoring. Their combination comprehensively covers all required precursor monitoring dimensions and sufficiently supports the core objective of this study regarding scenario adaptability.

The exclusion of other sensor types, such as hyperspectral and thermal infrared multispectral sensors, was primarily due to considerations of engineering feasibility and practicality. Firstly, such sensors involve high costs, with hardware and software investments significantly exceeding those of existing solutions, making them less compatible with the budget constraints of high-frequency and wide-coverage monitoring. Secondly, there are technical adaptability limitations: Hyperspectral sensors generate large volumes of data, impose stringent demands on UAV endurance and flight stability, and are susceptible to environmental interference. Meanwhile, the core functions of thermal infrared multispectral sensors can be achieved through the combination of thermal infrared imagers and multispectral sensors already included in this study, rendering redundant evaluation unnecessary. Additionally, the miniaturization and UAV integration technologies for these advanced sensors are still under development. They are currently more commonly used on satellite or manned aircraft platforms and are not yet mainstream options for routine engineering inspections.

2．**The analysis relies primarily on theoretical sensor performance metrics and flying height correction coefficients. Incorporating field data from actual UAV monitoring campaigns would provide more robust validation of the proposed method.**

Response: Thank you for your valuable suggestion. This study focuses on establishing a methodological framework for optimal sensor combination selection, with its core contribution lying in the development of an improved AHP-TOPSIS evaluation model and the validation of its scientific validity and universality. Therefore, at the current stage, validation is primarily conducted using theoretical metrics and height correction coefficients, with an emphasis on addressing the theoretical question of "how to scientifically select sensor types." As you rightly pointed out, integrating field data is a crucial direction to enhance the persuasiveness of validation and represents a limitation of this study. In response to your suggestion, we have specifically addressed this in the "Limitations" and "Future Research Directions" sections of the paper, explicitly identifying the conduct of systematic field validation as a key focus of subsequent work to further refine the reliability and applicability of the model.

3．**The paper mentions the need for exploring data fusion algorithms but does not delve into specific techniques. Discussing potential data fusion approaches and their impact on monitoring performance would be beneficial.**

Response: Thank you for your valuable suggestion, which we highly appreciate and have actively addressed. In the "Future Research Directions" section, we have added a dedicated subsection titled "Exploring Multi-sensor Data Fusion Technologies" (please refer to the revised manuscript for details). In this subsection, considering the characteristics of the sensor combinations screened in this study and the monitoring requirements of different highway scenarios, we have constructed a three- fusion technology framework encompassing data-level, feature-level, and decision-level fusion. Specifically:

To address the noise suppression requirements in surface distresses monitoring on rural level highways, data-level fusion techniques such as wavelet transform and Convolutional Neural Network (CNN)-based feature fusion are proposed.

To tackle the challenge of identifying deep-seated deformations under vegetation occlusion in rural rolling/rural mountainous areas, a feature-level fusion approach based on local feature matching techniques—including Principal Component Analysis (PCA) and Scale-Invariant Feature Transform (SIFT)—is introduced.

To enhance the reliability of monitoring conclusions in complex dynamic environments, decision-level fusion schemes utilizing Dempster-Shafer evidence theory and Bayesian inference are introduced.

This addition strengthens the paper's technical completeness and its guiding value for engineering applications.

4．**The study focuses on four specific highway areas. Discussing the generalizability of the results to other types of roads and regions would enhance the paper's value.**

Response: Thank you for your valuable suggestion, which we fully endorse and have actively addressed. We have added a new subsection titled "Extending Research Generalizability" in the "Future Research Directions" section of the paper (please refer to the revised manuscript for details). In this subsection, we first clarify the applicable scope of the study's conclusions, which encompasses four typical scenarios: rural level (plains), rural rolling (hills), rural mountainous (mountains), and urban/suburban areas. Subsequently, we emphasize the universality of the methodological framework, noting that for special road types such as those in deserts, permafrost regions, and coastal areas, despite differences in the "types of disaster precursors" and "weights of demand indicators", the underlying decision-making logic of "demand analysis → indicator quantification → solution optimization" remains universally applicable. Future research can extend this method to new scenarios by adjusting input parameters (e.g., tailoring indicators and weights to specific environmental characteristics) without reconstructing the model. This addition not only clarifies the application boundaries of the research but also highlights the potential for methodological generalization, effectively enhancing the rigor and foresight of the paper's conclusions.

**Reviewer's Review Comments**

1．Objectives of the study has not been mentioned anywhere in the paper. Add a separate section on the objectives of the study clearly stating the objectives of the study along with scope and limitations.

Response: Thank you for the valuable feedback. We have addressed this point by adding a clear and concise statement regarding the study's core objectives, application scope, and inherent limitations in the second paragraph of the Introduction section of the revised manuscript.

2．Add section on literature review before materials and methods section.

Response: Thank you for the suggestion. We have added a dedicated "Literature Review" section before the "Materials and Methods" section in accordance with your suggestion.

3．AHP has been used in the research so add the copy of the questionnaire in the annexure.

Response: Thank you for the suggestion. We have added a "Questionnaire" appendix to the paper, which is the expert evaluation questionnaire used for the Analytic Hierarchy Process (AHP) in this study.

4．Support conclusions with data from analysis and results sections.

Response: Thank you for the suggestion. We have revised the Conclusions section accordingly to ensure that all stated conclusions are directly supported by the data and results from the Analysis and Results section of the paper.
