# Supplementary material for: Research on multi-sensor fusion architecture for highway area hazard monitoring based on UAVs
Source: PLoS One. 2026 May 7;21(5):e0348241. doi: 10.1371/journal.pone.0348241 (PMC13152189; doi:10.1371/journal.pone.0348241)
Supplement: Attachment — Submitted filename: Response to Reviewers(2).docx [file pone.0348241.s003.docx]

**Response to Reviewers**

1．**The study focuses on five common sensors, but there are several other sensors available for UAV-based monitoring (e.g., hyperspectral, thermal infrared multispectral). A broader evaluation of sensor options would strengthen the study.**

Response: Thank you for the suggestion. We have expanded our discussion to incorporate hyperspectral and thermal infrared multispectral sensors.

2．**The analysis relies primarily on theoretical sensor performance metrics and flying height correction coefficients. Incorporating field data from actual UAV monitoring campaigns would provide more robust validation of the proposed method.**

Response: Thank you for the suggestion. In Section 4.4 of the paper, we have added experimental results from existing literature based on field-collected UAV data, providing empirical support for the proposed optimal fusion scheme.

3．**The paper mentions the need for exploring data fusion algorithms but does not delve into specific techniques. Discussing potential data fusion approaches and their impact on monitoring performance would be beneficial.**

Response: Thank you for the suggestion. We have added a discussion on specific potential data fusion methods and their impact on monitoring performance in Section 4.5 of the paper.

4．**The study focuses on four specific highway areas. Discussing the generalizability of the results to other types of roads and regions would enhance the paper's value.**

Response: Thank you for the suggestion. We have added a discussion in Section 3.5 of the paper regarding the potential generalizability of the findings to other road types and regions.
